# Engineered Lysin-Derived Peptide as a Potent Antimicrobial for Acne Vulgaris

**DOI:** 10.3390/antibiotics14040344

**Published:** 2025-03-27

**Authors:** Uri Sela, Ryan D. Heselpoth, Vincent A. Fischetti

**Affiliations:** Laboratory of Bacterial Pathogenesis and Immunology, The Rockefeller University, 1230 York Avenue, New York, NY 10065, USA; rheselpoth@rockefeller.edu (R.D.H.); vaf@rockefeller.edu (V.A.F.)

**Keywords:** Acne vulgaris, lysin, lysin-derived peptide, cationic peptide, antimicrobial, *Cutibacterium acnes*, *Staphylococcus aureus*, therapeutic, bactericidal, bacteriophage

## Abstract

**Background/Objectives**: Acne vulgaris is a skin disorder that affects millions worldwide, with *Cutibacterium acnes* playing a key role in its inflammation. Antibiotics reduce *C. acnes* and inflammation, but growing antibiotic resistance has limited their efficacy. Additionally, other common acne treatments with bactericidal activity, like benzoyl peroxide, cause irritation, dryness, and peeling. To fulfill the unmet need for alternative therapies, our strategy focused on identifying potent phage lysins and/or their derived cationic peptides. **Methods**: The C-terminal cationic antimicrobial peptide of the *Prevotella intermedia* phage lysin PlyPi01 was synthesized along with several sequence-engineered variants in an attempt to enhance their bactericidal efficacy. In vitro bacterial killing assays evaluated the potency of the lysin-derived peptide derivatives against *C. acnes* and *Staphylococcus aureus*, another skin bacterium associated with acne. Antibacterial activity was assessed both in conditions simulating the human skin and in combination with retinoids. **Results**: The variant peptide P156 was engineered by adding arginine residues at both the N- and C-terminal ends of the parental peptide PiP01. P156 was highly potent and eradicated all tested strains of *C. acnes* and *S. aureus*. P156 acted rapidly (>5-log kill in 10 min), further reducing the potential of resistance development. Additionally, P156 maintained its potency under conditions (e.g., temperature, pH, and salt concentration) observed on the skin surface and in hair follicles, as well as in combination with retinoid—all without being toxic to human cells. **Conclusions**: These collective findings position P156 as a promising topical drug for clinical applications to control acne vulgaris.

## 1. Introduction

Acne vulgaris is a very common skin problem, affecting most adolescents worldwide, and, in some cases, persisting into adulthood. Globally, acne vulgaris ranks eighth in overall disease prevalence [1]. Acne is a chronic inflammatory disease of the skin pilosebaceous unit that produces sebum to lubricate both the skin and hair, and acts as a natural barrier against external environmental factors [1,2]. While the pathogenesis of acne is multifactorial, dysbiosis of the skin microbiome is an important factor, and the bacterium *Cutibacterium acnes* (*C. acnes*, formerly *Propionibacterium acnes*) plays a key role [3]. Mechanistically, *C. acnes* contributes to the inflammatory process that is typical of acne vulgaris, and therefore, the elimination of *C. acnes* is part of current therapeutic protocols [1,2,4,5,6,7,8]. In some cases, other skin microbiome members, such as staphylococci, including *Staphylococcus aureus* (*S. aureus*), can contribute to skin inflammation [3]. Moreover, *S. aureus* is one of several microbes that can add to the imbalances found in the skin microbiome described in acne vulgaris [9].

*C. acnes* is a lipophilic Gram-positive bacterium [10]. While *C. acnes* grows ideally in anaerobic lipid-rich conditions, it is also an aerotolerant bacterium that can detoxify oxygen and, therefore, can be sustained on the surface of the skin [11]. Although considered a commensal, *C. acnes* involvement in various infections—e.g., bone and prosthesis, spinal disk, eyes after cataract surgery, central nervous system catheters, and others—led to its emergence as an opportunistic pathogen [10].

Current protocols for the treatment of acne vulgaris include the use of antibiotics to eradicate *C. acnes*, thereby mitigating the typical inflammatory process in acne lesions [3,7]. These antibiotics are often combined with topical benzoyl peroxide or retinoids to further mitigate inflammation [7]. Due to concerns about the development of antibiotic resistance, current acne treatment guidelines recommend limiting antibiotic use to a duration of up to 3 months. However, in clinical practice, the length of antibiotic treatment is often significantly longer, and as such, the prevalence of *C. acnes* strains that are resistant to various antibiotics is increasing [12,13,14,15]. Taken together, the need for treatment alternatives is evident.

Lysins are bacteriophage (phage)-encoded enzymes used by the phage to degrade the bacterial cell wall and promote hypotonic lysis, thereby releasing progeny virions from the phage-infected cells. When delivered externally as a purified recombinant protein, lysins may be used as efficient antimicrobials to rapidly lyse the target bacteria [16]. Native lysins that kill Gram-positive (G+) pathogens degrade the peptidoglycan by means of hydrolyzing critical covalent bonds in the structure, while lysins directed to Gram-negative (G−) bacteria must initially disrupt the outer membrane to subsequently access and degrade the peptidoglycan. Because of this dual action, most native G− lysins structurally comprise a single globular peptidoglycan-degrading catalytic domain with a C-terminal cationic region responsible for destabilizing the outer membrane, possibly through competitive displacement of stabilizing divalent cations located on the surface of G− bacteria [17]. We found that when the C-terminal cationic segment is isolated and delivered externally on its own as a peptide, it kills the G− bacteria by destabilizing both the outer and cytoplasmic membranes [18,19]. Further, we demonstrated that the latter effect enables strong activity against G+ bacteria as well [19]. 

When compared to small molecule antibiotics, lysins have several innate characteristics that make them advantageous as potential antibacterial therapeutics. Lysins display rapid bacterial killing kinetics and can disrupt biofilms, with the latter often resistant to antibiotics [20]. For certain G+ lysins, their antibacterial activity is narrow-spectrum due to their high specificity, thus decreasing side effects often associated with the disruption of the gut flora by broad-spectrum antibiotics. Importantly, resistance to lysins has not been observed to date. This is most likely attributable to their aforementioned intrinsic properties, as well as the fact that these enzymes target and degrade the highly immutable bacterial cell wall peptidoglycan [16]. Alternatively, while lysin-derived peptides appear to display broad-spectrum antibacterial activity and thus lack the specificity of G+ lysins, these peptides effectively kill antibiotic-resistant bacteria on contact and are highly thermostable [19].

Currently, as far as we know, there is a lack of effective lysins for therapeutic use against *C. acnes* exhibiting >3-log colony forming units (CFU) reduction. Furthermore, no lysin-derived peptide has been shown to be active against *C. acnes*. In this study, we modified a lysin-derived peptide from a *Prevotella intermedia* phage by strategically introducing cationic amino acids to greatly improve its bactericidal activity against bacterial species associated with acne vulgaris, namely *C. acnes* and *S. aureus.*

## 2. Results

### 2.1. Modified PlyPi01-Derived Peptides Display Potent Bactericidal Activity

**Figure 1:**
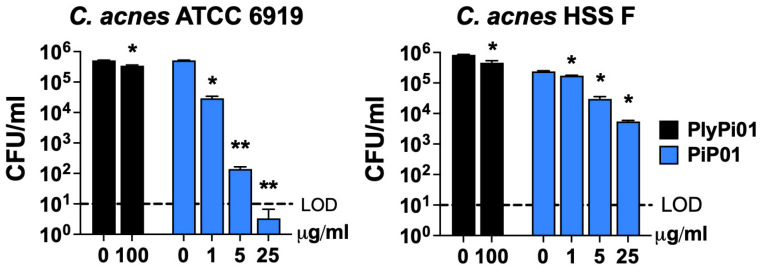
**Antibacterial activity of lysin PlyPi01 and peptide PiP01 against *C. acnes*.** The antibacterial activity of the PlyPi01 lysin was compared to its isolated C-terminal cationic peptide, PiP01. Bactericidal activity was measured against *C. acnes* strains ATCC 6919 (**left**) and HSS F (**right**) in 20 mM Tris, pH 7.2, for 1 h at 37 °C. Bacterial viability was assessed via serial dilution and plating. The limit of detection (LOD, dashed lines) was 10 CFU/mL. Bactericidal activity is defined as a ≥3-log CFU reduction with respect to the untreated control. Error bars represent the ±SEM from triplicate experiments. *p-*values were calculated using an unpaired *t*-test between each peptide or lysin dose and the untreated group. * *p* < 0.05 and a <3-log CFU reduction, ** *p* < 0.05 and a ≥3-log CFU reduction.

**Figure 2:**
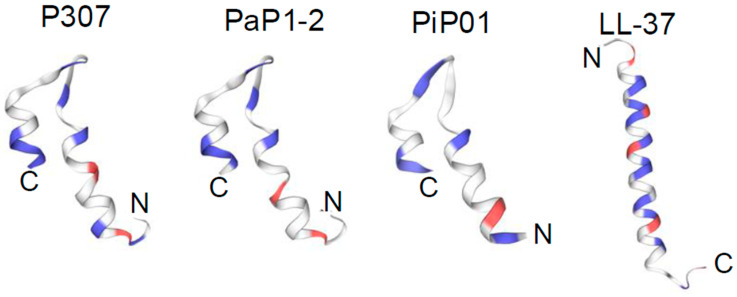
**Predicted structure of the parental lysin-derived peptide PiP01.** SWISS-MODEL was used to compare the predicted structure of the lysin-derived peptide PiP01 to that of the lysin-derived peptides P307 and PaP1-2. All three peptides are predicted to adopt comparable helix–loop–helix hairpin structural motifs. For comparison, the alpha-helical structure of the human AMP LL-37 is also shown. Blue, positively charged amino acids; red, negatively charged amino acids.

To identify a potential lysin or lysin-derived peptide with activity against *C. acnes*, a bioinformatic search was restricted to anaerobic G− bacteria with <50% homology to our reported G− lysin PlyF307 from *Acinetobacter baumannii* [21]. The potential candidate lysin also had to comprise a putative C-terminal cationic region with comparable physicochemical characteristics to that of PlyF307-derived peptide P307 [18], including length, charge, hydropathicity, and predicted structure. Ultimately, a 141 aa lysin, PlyPi01, from a *Prevotella intermedia* lysogen (GenBank: MBQ0073608.1) with a predicted single globular muramidase domain was identified, expressed, and purified. However, this whole lysin (PlyPi01) exhibited low antibacterial activity (<0.5-log kill) against *C. acnes* (Figure 1). We then synthesized the C-terminal positively charged region of PlyPi01 (aa 102–132), termed PiP01 (31 aa peptide, Table 1). The predicted helix–loop–helix hairpin structure of PiP01 was similar to other lysin-derived peptides, such as P307 and PaP1-2 (Figure 2), but showed less resemblance to the human cationic antimicrobial peptide LL37 [18,19]. While the PiP01 peptide was bactericidal at ≥5 μg/mL when tested against *C. acnes* strain ATCC 6919, its activity was only modest against *C. acnes* strain HSS F (Figure 1).

**Figure 3:**
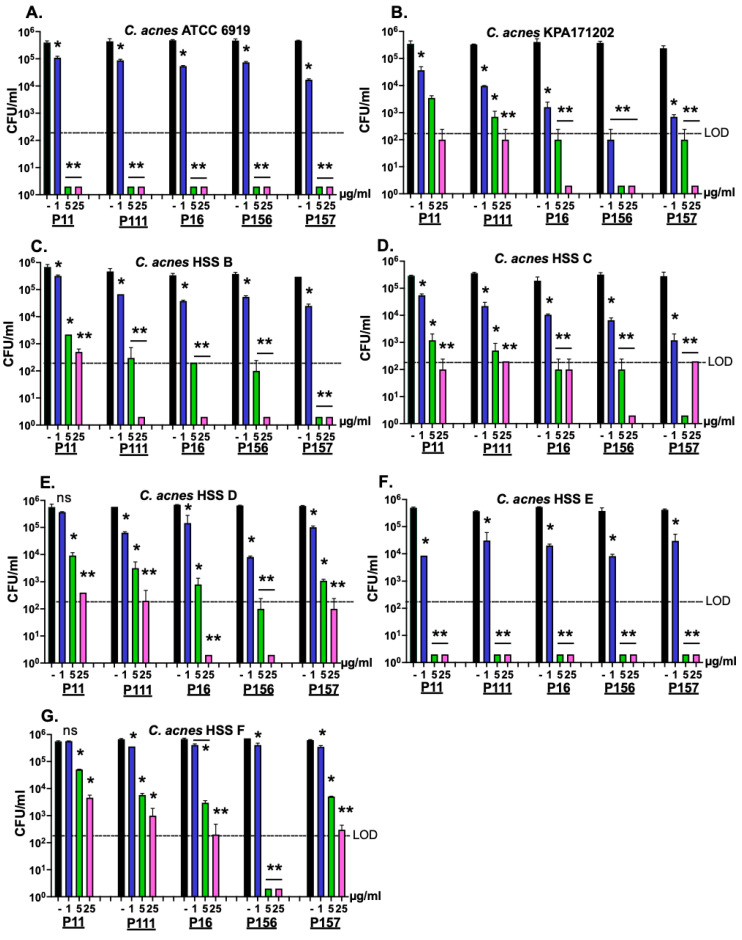
**Bactericidal activity of five modified peptides derived from the *P. intermedia* PlyPi01 lysin on various strains of *C. acnes*.** The amino acid sequence pertaining to the cationic C-terminal region of PlyPi01 (aa 102–132) was isolated and strategically engineered with varying cationic aa modifications. Using a dose-response killing assay, a total of five peptide derivatives (P11, P111, P16, P156, and P157) were assayed for bactericidal activity against the *C. acnes* clinical isolates (**A**) ATCC 6919, (**B**) KPA171202, (**C**) HSS B, (**D**) HSS C, (**E**) HSS D, (**F**) HSS E and (**G**) HSS F. Following 1 h treatment in 20 mM Tris, pH 7.2, at 37 °C, bacterial viability was assessed via serial dilution and plating. The limit of detection (LOD, dashed lines) was 200 CFU/mL. Bactericidal activity is defined as a ≥3-log CFU reduction with respect to the untreated control. Error bars represent the ±SEM from duplicate experiments. *p-*values were calculated using an unpaired *t*-test between each peptide dose and untreated group. ns, not significant, * *p* < 0.05 and a <3-log CFU reduction, ** *p* < 0.05 and a ≥3-log CFU reduction.

Modifying the positive net charge of antimicrobial peptides has been previously shown to greatly enhance its antimicrobial activity [22]. Therefore, to improve the bactericidal activity of PiP01 to cover most, if not all, strains of *C. acnes*, five modified versions of the peptide (P11, P111, P16, P156, and P157) were synthesized with the random addition of positively charged amino acids (lysine and arginine) to the N- and/or C-terminal ends. The bactericidal potency of each peptide was evaluated against several clinical isolates of *C. acnes* (Table 2) using a dose–response killing assay. As shown in Figure 3, when assayed at ≥5 μg/mL, each peptide was capable of more than one log reduction in CFU counts of all *C. acnes* strains except for the HSS F strain. Compared to the other modified peptides, P156 exhibited the highest bactericidal potency, as P156 was the only peptide capable of lowering the viability of all *C. acnes* strains below the limit of detection (LOD) at ≥5 μg/mL.

**Figure 4:**
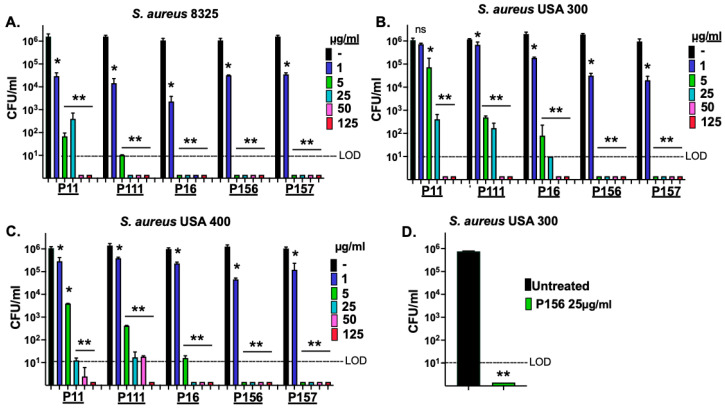
**P11, P111, P16, P156, and P157 antibacterial activity against MSSA and MRSA strains of *S. aureus*.** (**A**) MSSA strain 8325, (**B**) MRSA strain USA 300, and (**C**) MRSA strain USA 400 were grown under aerobic conditions to the mid-log phase. A dose–response killing assay was performed to assess the bactericidal activity of P11, P111, P16, P156, and P157 against each *S. aureus* strain in 20 mM Tris, pH 7.2, for 1 h at 37 °C. (**D**) The MRSA strain USA 300 was grown under anaerobic conditions to mid-log phase and then treated with P156 at 25 μg/mL in 20 mM Tris, pH 7.2, for 1 h at 37 °C. Bacterial viability was quantified via serial dilution and plating. The limit of detection (LOD, dashed lines) was 10 CFU/mL. Bactericidal activity is defined as a ≥3-log CFU reduction with respect to the untreated control. Error bars represent the ±SEM from triplicate experiments. *p-*values were calculated using an unpaired *t*-test between each peptide dose and the untreated group. ns, not significant, * *p* < 0.05 and a <3-log CFU reduction, ** *p* < 0.05 and a ≥3-log CFU reduction.

*S. aureus* constitutes a major component of the skin microbiome and can contribute to the inflammation of acne vulgaris. It colonizes the skin surface under aerobic conditions but can expand into the hair follicle, where anaerobic conditions may predominate [28]. To evaluate the bactericidal activity of the peptide derivatives against *S. aureus*, we used standard methicillin-resistant *S. aureus* (MRSA) strains USA300 and USA400 (Table 2), as well as the methicillin-sensitive *S. aureus* (MSSA) strain 8325 in a dose–response killing assay. Under aerobic conditions (Figure 4A–C), all five peptide derivatives at 50 μg/mL demonstrated strong antimicrobial activity against the MRSA and MSSA strains, with >5-log CFU reduction, dropping counts to or below the LOD (10 CFU/mL). However, P156 and P157 could also reduce CFU counts for all the *S. aureus* strains below the LOD at concentrations as low as 5 μg/mL.

Taken together, the results from Figure 3 and Figure 4 demonstrate that only P156 at 5 μg/mL was bactericidal (≥3-log kill) against all *C. acnes* and *S. aureus* strains tested. Additionally, at 25 μg/mL, it was the only peptide capable of lowering the viability of all the bacterial strains below the LOD (>4-log CFU reduction). The sequence and properties of the selected best peptide, P156, are shown in Table 1.

Since *S. aureus* growth may also occur within the hair follicle, we next aimed to study whether P156 can also exhibit activity against *S. aureus* grown under anaerobic conditions. As shown in Figure 4D, like the results observed under aerobic growth conditions, P156 at 25 μg/mL was bactericidal when tested against MRSA strain USA300 under anaerobic conditions, reducing CFU counts to below the LOD, resulting in >5-log kill.

### 2.2. P156 Kills C. acnes Under Conditions Relevant to Skin Properties and Bacterial Growth

**Figure 5:**
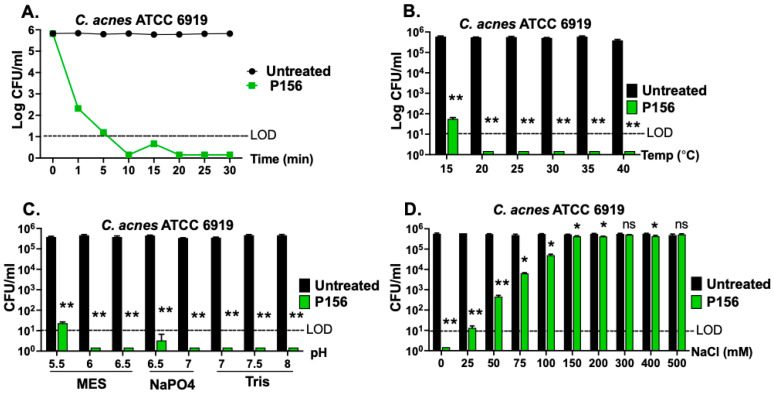
**Bactericidal properties of P156 under various relevant skin conditions.** (**A**) The killing kinetics of P156 (at 25 μg/mL) against *C. acnes* strain ATCC 6919 (in 20 mM Tris, pH 7.2, at 37 °C) was assessed over 30 min at various time points by measuring bacterial viability. Significance level for all P156-treated samples at ≥1 min was **. (**B**–**D**) The bactericidal activity of P156 (at 25 μg/mL) was quantitated at various (**B**) temperatures (15–40 °C), (**C**) pH values (20 mM MES: pH 5.5–6.5; 20 mM NaPO_4_: pH 6.5–7.0; 20 mM Tris, pH 7.0–8.0) or (**D**) NaCl concentrations (0–200 mM). The limit of detection (LOD, dashed lines) was 10 CFU/mL. Bactericidal activity is defined as a ≥3-log CFU reduction with respect to the untreated control. Error bars represent the ±SEM from triplicate experiments. *p-*values were calculated using an unpaired *t*-test between each peptide dose and the untreated group. ns, not significant, * *p* < 0.05 and a <3-log CFU reduction, ** *p* < 0.05 and a ≥3-log CFU reduction.

An effective bactericidal agent for treating acne vulgaris should rapidly eliminate *C. acnes* upon skin application. Therefore, we evaluated the time–kill kinetics of P156 against *C. acnes*. As shown in Figure 5A, P156 demonstrated rapid action, achieving >3-log reduction in bacterial CFU within 1 min and >5-log reduction in 10 min at 25 μg/mL.

Additionally, the bactericidal activity of P156 was evaluated under conditions relevant to skin properties and optimal *C. acnes* growth. The human skin surface temperature typically ranges from 32 °C to 37 °C, close to the optimal growth temperature for *C. acnes* of 30 °C to 37 °C [29]. To simulate these conditions, P156 was thawed from −80 °C and stored for four weeks at 4 °C, and its bactericidal activity was assessed at various temperatures. Figure 5B shows that the peptide at 25 μg/mL maintained potent bactericidal activity across a broad range of temperatures (20 °C to 40 °C), reducing *C. acnes* CFU counts below the LOD (>5 logs). This finding also suggests that the peptide remains stable and active even after four weeks of storage at 4 °C.

The optimal pH for *C. acnes* growth ranges from 6.0 to 7.0, which is within the pH range found in hair follicles (6.3 to 6.6) [30,31]. However, the normal skin surface itself also tends to be slightly more acidic [31]. P156, at 25 μg/mL, exhibited significant bactericidal activity across a broad pH range (5.5 to 8.0), achieving >4-log CFU reduction (Figure 5C).

Next, NaCl concentration was considered in relation to conditions of sweat on the skin surface. While NaCl levels on the skin in the absence of sweat are very low (<1 mM), concentrations within sweat glands can, on average, reach up to 50 mM [32]. Although NaCl levels on sweat gland-adjacent skin or within hair follicles under sweat conditions are less known, considering the diffusion of sweat from sweat glands to these adjacent areas, they are estimated to range between 10 and 50 mM. Figure 5D illustrates that P156, at 25 μg/mL, achieved a reduction of >3-log CFU counts at ≤50 mM NaCl and a reduction to below the LOD (>4-log CFU) at a concentration under 25 mM NaCl.

### 2.3. P156 Lacks Cytotoxicity Toward Human Cells

**Figure 6:**
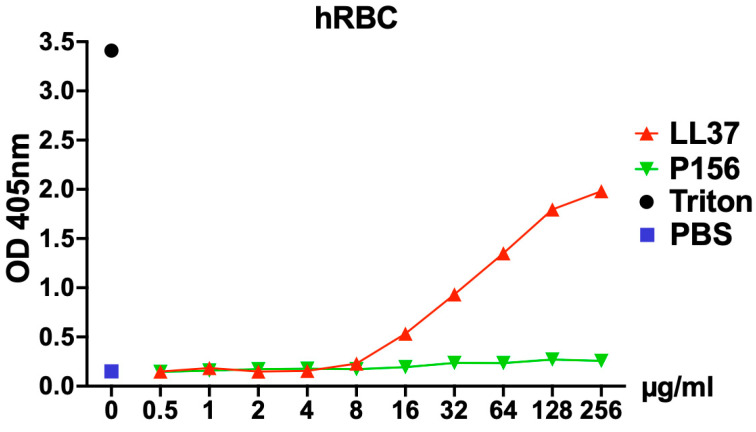
**P156 cytotoxicity toward eukaryotic cells.** hRBCs from healthy donors were incubated for 4 h at 37 °C in 5% CO_2_ with either PBS (negative control, blue square), 0.1% Triton X-100 (positive control, black dot), P156 (green triangle), or LL37 (a human antimicrobial peptide used as a control, red triangle). For the peptide treatments, a concentration gradient ranging from 0.5–256 μg/mL in PBS was applied. After the incubation, intact hRBCs were removed, and the relative concentration of hemoglobin released into the supernatant was quantified by measuring the absorbance at 405 nm. Data shown represent one of three independent experiments with similar results.

To assess the cytotoxicity of P156 on human cells, hemolysis of human red blood cells (hRBCs) was evaluated after incubation with a wide concentration range of P156 (0.5–256 μg/mL) in phosphate-buffered saline (PBS). The human cationic AMP LL-37 was used as a positive control due to its known potential for inducing hRBC toxicity (hemolysis) [33]. As seen in Figure 6, LL-37 exhibited a dose-dependent increase in optical density at 405 nm (OD_405nm_), indicating progressive hemolysis. In contrast, P156 showed no hRBC toxicity and maintained a consistent OD value across all peptide concentrations tested, comparable to the PBS negative control.

### 2.4. P156 Bactericidal Activity Is Preserved When Used in Combination with Current Common Acne Vulgaris Treatments

**Figure 7:**
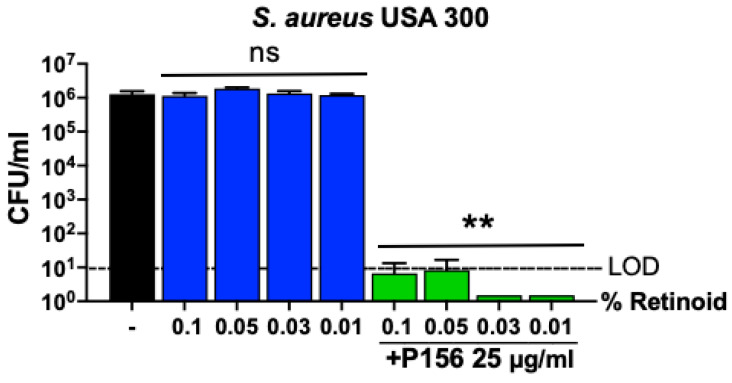
**Effect of retinoids on P156 bactericidal activity.** Retinoic acid at various concentrations (0.01%, 0.03%, 0.05%, 0.1%) was incubated with MRSA USA300 either alone or in combination with P156 (25 μg/mL) during a 1 h killing assay in Tris, pH 7.2, at 37 °C. Bacterial viability was assessed by serial dilution and plating. The limit of detection (LOD, dashed lines) was 10 CFU/mL. Bactericidal activity is defined as a ≥3-log CFU reduction with respect to the untreated control. Error bars represent the ±SEM from triplicate experiments. *p-*values were calculated using an unpaired *t*-test between each peptide dose and the untreated group. ns, not significant, ** *p* < 0.05 and a ≥3-log CFU reduction.

In addition to antibiotics to eradicate *C. acnes*, accepted treatment protocols for acne vulgaris include retinoids. The guidelines of the American Academy of Dermatology for the treatment of acne vulgaris indicate that retinoids are the core of topical therapy for acne due to their comedolytic and anti-inflammatory activity [34]. Since retinoids are often used alongside antibiotics, the bactericidal activity of P156 was tested in combination with a retinoid to rule out any potential antagonism. Treating *S. aureus* for 1 h with retinoic acid alone, at concentrations typically used for acne vulgaris treatment (0.01–0.1%), exhibited no antibacterial effect (Figure 7). Conversely, in the presence of 0.01–0.1% retinoic acid, P156 (at 25 μg/mL) lowered *S. aureus* viability below the LOD (>5-log CFU reduction). This indicates that the bactericidal potency of P156 remains unaffected when used in combination with retinoic acid.

## 3. Discussion

Current therapeutic protocols for acne vulgaris aim to mitigate inflammation by eradicating the bacterial stimulus. However, prolonged antibiotic use has shown the development of resistance. In this paper, we evaluated in vitro the potential clinical use of a lysin-derived peptide as an antibiotic alternative to eradicate and control *C. acnes* on the skin surface, a major contributor to the pathogenesis of acne.

We initially tested the whole purified PlyPi01 lysin from a lysogen found in *P. intermedia* for antibacterial activity against *C. acnes* and found it had a minimal effect (<0.5-log kill, Figure 1). As an alternative strategy, the native C-terminal cationic peptide derived from PlyPi01 (aa 102–132), PiP01, was identified, isolated, and tested for activity (Figure 1). The modest antibacterial potency of the PiP01 peptide was enhanced using an engineering strategy outlined by a previous publication [22]. We randomly added the positively charged amino acid arginine to a number of synthesized PiP01 peptides, and five of these modified peptides displayed improved bactericidal properties against *C. acnes*. For each modified peptide, bactericidal potency was dose- and, except for P156, strain-dependent (Figure 3). P156 notably demonstrated a strong bactericidal activity (≥3-log kill) against all seven of the *C. acnes* strains tested, as well as *S. aureus*, at concentrations as low as 5 μg/mL, and >4-log kill at 25 μg/mL (Figure 3 and Figure 4). As a result, we used this specific peptide to evaluate its ability to kill *C. acnes* under conditions (pH, temperature, and NaCl) found in normal human skin.

Although speculative, the proposed antibacterial mechanism of action for P156 was formulated in accordance with how other cationic antimicrobial peptides function [35,36,37]. We hypothesize that P156 initially electrostatically interacts with the anionic bacterial cell surface, specifically through the negatively charged phosphate groups found in the cell wall and abundantly present in the bacterial cell membrane. This results in the competitive displacement of stabilizing divalent cations, which consequently distorts and weakens the structural integrity of the membrane to increase permeability. The balance of positively charged and hydrophobic amino acids permits P156 to adopt an amphipathic conformation, resulting in the several-fold accumulation of the peptide on the bacterial surface via electrostatics, followed by its subsequent insertion into the bacterial membrane to disrupt its structure.

To better predict its success in future human clinical trials, P156 should ideally be evaluated in an animal model. However, the absence of effective animal models for acne vulgaris presents a significant challenge in dermatological research [38,39]. This is primarily due to key biological differences (e.g., structure of sebaceous glands and hair follicles and variation in the microbiome) between humans and animals, particularly mouse models, which fail to accurately mimic the condition of acne vulgaris. Due to these limitations and considering that treatment would occur on the skin surface rather than systemically, we instead evaluated the bactericidal activity of P156 in vitro under conditions that closely simulate human skin and hair follicles. The experiments were carried out under optimal conditions for *C. acnes* growth. In these experiments, we aimed to better approximate the real environment for therapeutic intervention.

A major clinical challenge with current antibiotic treatments for acne vulgaris is the development of bacterial resistance. A rapid-acting bactericidal agent, such as P156, can reduce the likelihood of resistance by quickly eliminating the bacteria before they are able to adapt to the drug. Rapid action also helps resolve inflammation quickly and prevent the formation or worsening of acne lesions. P156 appears to kill *C. acnes* on contact, killing >3-logs within one minute and >5 logs in ten minutes, making it useful in a clinical setting (Figure 5A).

Additionally, P156 maintained its bactericidal efficacy within the optimal temperature range for *C. acnes* growth (30 °C to 37 °C) and across a broad range of possible facial skin temperatures, reducing bacterial counts >4 logs at each temperature assayed (Figure 5B). P156’s stability, shown by its activity after thawing from −80 °C to 4 °C and 4-week storage at this temperature, suggests a long shelf life and high efficacy under practical conditions.

The activity of P156 was also quantitated against *C. acnes* at physiological pH and salt. The optimal pH range for *C. acnes* growth (pH 6–7) is similar to the pH found within hair follicles, while the normal skin surface tends to be more acidic. This suggests that *C. acnes* growth on the skin surface might be slower in more acidic, aerobic conditions. P156 exhibited a robust bactericidal effect, achieving a ≥4-log CFU reduction across a broad pH range, with complete bacterial eradication within the optimal pH range for *C. acnes* growth (pH 6–7). However, in patients with acne vulgaris, skin pH is often higher (up to 7.6 in [40]). Despite this, P156 was able to eradicate *C. acnes* at these elevated pH levels. In addition to pH, we measured the effect of salt on the antibacterial activity of P156. The salt concentration in the skin and hair follicles is affected by sweat. Within NaCl levels typical for the skin—ranging from less than 1 mM in the absence of sweat to up to 50 mM with sweat—the peptide effectively reduced *C. acnes* populations by 3-logs in 50 mM NaCl. Moreover, the killing efficiency of P156 improved significantly (>4 logs) at lower salt concentrations (Figure 5D). The lack of antibacterial activity displayed by P156 at high salt concentrations is most likely due to the salt neutralizing the cationic charge properties of the peptide, thus inhibiting the initial electrostatic interaction required between the cationic peptide and the negatively charged bacterial surface for antibacterial activity. As previously reported with the lysin-derived PaP1 peptide, the salt sensitivity of P156 could potentially be reduced by end-tagging the peptide with short hydrophobic oligopeptides [19].

The mechanism of action of cationic AMPs has been previously described and includes the destabilization of bacterial cell membranes [38]. Because the cytoplasmic membrane of G+ bacteria, such as *C. acnes* and *S. aureus*, share similarities with human cell membranes (i.e., phospholipid bilayer), and because other AMPs (e.g., LL-37 in Figure 6 and ref. [33], or colistin in ref. [41]) have demonstrated cytotoxicity to human cells, it was crucial to rule out potential human cell toxicity associated with P156. Results from a hemolytic assay revealed that, similar to the PBS negative control and unlike LL-37, P156 does not disrupt the membrane of hRBCs (Figure 6). Like other lysin-derived peptides [18,19], the lack of cytotoxicity associated with P156 indicates that the peptide exhibits selective activity toward prokaryotic membranes.

Drug combinations are often essential in the treatment of acne vulgaris due to the multifactorial nature of the condition. Acne results from a combination of factors like excess sebum production, bacterial colonization (particularly *C. acnes*), inflammation, and abnormal keratinization of skin cells. Using multiple drugs targeting different aspects of acne can increase treatment efficacy and reduce the risk of resistance or side effects. The current therapeutic protocols include a combination of antibiotics with at least one anti-inflammatory component, most commonly retinoid. In this regard, P156 maintained its activity when combined with retinoids (Figure 7) and, as such, may be an effective addition to the treatment of acne vulgaris.

## 4. Material and Methods

### 4.1. Bacterial Strains and Culture Conditions

Bacterial strains used in this study are outlined in Table 2 and were stored at −80 °C. *C. acnes* strains were grown in a BACTRON900 anaerobic chamber (Sheldon Manufacturing Inc., Cornelius, OR, USA) at 37 °C to exponential phase in reduced Trypticase Soy Broth (rTSB, BD Biosciences, Franklin Lakes, NJ, USA) supplemented with 2% (*v*/*v*) glycerol. *S. aureus* strains were grown either aerobically or anaerobically at 37 °C to the exponential phase in TSB (aerobic growth) or rTSB (anaerobic growth).

### 4.2. PlyPi01 Lysin Protein Expression and Purification

Using the expression construct *Escherichia coli* BL21(DE3) pET28a::*plyPi01*, the PlyPi01 lysin was expressed in Luria–Bertani medium (BD Biosciences) supplemented with 50 μg/mL kanamycin (Fisher Scientific, Waltham, MA, USA) at 18 °C for 16–18 h. Protein expression was induced at mid-log phase (OD_600nm_ = 0.5) with 1 mM isopropyl β-D-1-thiogalactopyranoside (Biosynth, Ystad, Switzerland). The bacteria were resuspended in 50 mM Tris-HCl (Fisher Scientific), pH 7.5, 200 mM NaCl (Fisher Scientific), and 1 mM phenylmethylsulfonyl fluoride (Sigma-Aldrich, St. Louis, MO, USA) and then subsequently lysed using a Q125 sonicator (QSonica, Newtown, CT, USA). The whole cell lysate was clarified by centrifugation at 12,000 RPM for 1 h at 4 °C. The soluble lysate fraction was passed through a 0.2 μm syringe filter and then dialyzed into 10 mM sodium phosphate, pH 7.0 (Fisher Scientific). Next, the protein sample was applied to a 5 mL HiTrap SP FF column (Cytiva, Marlborough, MA, USA) in 10 mM sodium phosphate, pH 7.0, at 2 mL/min. PlyPi01 was eluted from the column using a 20 CV linear gradient from 0-500 mM NaCl. Purification fractions comprising pure PlyPi01 were pooled and dialyzed overnight against PBS, pH 7.4. PlyPi01 was further purified using a HiLoad 16/600 Superdex 200 PG column (Cytiva). The protein was applied to the column at 1 mL/min in PBS, pH 7.4. Elution fractions consisting of highly pure PlyPi01 were combined, concentrated, supplemented with 10% (*v*/*v*) glycerol (Fisher Scientific), and then passed through a 0.2 μm syringe filter. Aliquots of the purified lysin were stored at −80 °C until further needed.

### 4.3. Peptide Synthesis and Properties

The various engineered peptides were synthesized by Biomatik Corporation (Wilmington, DE, USA). The structures of various peptides were predicted using SWISS-MODEL (https://swissmodel.expasy.org, accessed on 10 February 2025) [42]. P156 properties (Table 1) were predicted with ProtParam-Expasy (https://web.expasy.org/protparam/, accessed on 10 February 2025). Net charge at pH 7.4 was calculated using the Prot pi-Protein tool, version 2.2.29.152.

### 4.4. One-Hour Killing Assays

Unless stated otherwise, the standard conditions for killing assays involved statically treating exponential phase bacteria at approximately 5 × 10^5^ CFU/mL with a lysin-derived peptide at 25 μg/mL in a 96-well microtiter plate under aerobic (*S. aureus*) or anaerobic conditions (*C. acnes* and *S. aureus*) in 20 mM Tris (Fisher Scientific), pH 7.2, for 1 h at 37 °C. For dose–response killing assays, peptide concentrations ranging from 1 up to 125 μg/mL were tested.

To evaluate the effect salt has on the antibacterial activity of the peptide, *C. acnes* strain ATCC 6919 was treated with P156 in buffer supplemented with 0-500 mM NaCl. The bactericidal potency of P156 in a broad pH range was assayed by treating *C. acnes* strain ATCC 6919 with the peptide in either 20 mM 2-(N-morpholino) ethanesulfonic acid (MES, pH 5.5–6.5; Fisher Scientific), sodium phosphate buffer (NaPO_4_, pH 6.5–7.0), or Tris (pH 7.0–8.0).

Temperature-based killing assays were performed by initially thawing a frozen aliquot of P156, which was then stored at 4 °C for a month. This was the only experiment where an aliquot of a lysin-derived peptide was stored for an extended period of time at a temperature other than −80 °C. Next, *C. acnes* strain ATCC 6919 was treated with the above-mentioned P156 aliquot in buffer at 15 °C to 40 °C. For these experiments, separate aliquots of both the bacteria at 10^6^ CFU/mL and peptide at 50 μg/mL were initially equilibrated to the temperature being assayed for 5 min in 1.7 mL microcentrifuge tubes using an EchoTherm chilling/heating plate (Torrey Pines Scientific, Carlsbad, CA, USA). The bacteria and peptide were then mixed together 1:1 in a microcentrifuge tube, yielding respective final concentrations of 5 × 10^5^ CFU/mL and 25 μg/mL, and subsequently incubated in the chilling/heating plate for 1 h.

For measuring the effect of retinoid on peptide bactericidal activity, *S. aureus* strain USA300 at 10^6^ CFU/mL was treated with or without P156 at 25 μg/mL in buffer supplemented with all-trans-retinoic acid (Sigma-Aldrich) at 0.01–0.1% (*w*/*v*).

Each killing assay comprised an untreated control (bacteria absent peptide) for every condition tested. After 1 h, 100 μL directly from the sample, as well as 5 μL of each dilution from a 10-fold serial dilution, were plated on either TSB agar (for *S. aureus* killing assays using aerobic conditions), rTSB agar (for *S. aureus* killing assays using anaerobic conditions), or rTSB agar with 2% glycerol (for *C. acnes* killing assays) in order to quantitate bacterial viability. The LOD for each experiment was 10 CFU/mL, with one exception. For the dose–response killing assay evaluating the collection of peptides against *C. acnes*, only the 10-fold serial dilutions from each sample were plated, resulting in an LOD of 200 CFU/mL. Experiments performed under anaerobic conditions used agar plates that were reduced overnight. Additionally, buffers for these particular assays were made fresh the day of the experiment and reduced for 2 h prior to use. Error bars correspond to the standard error of the mean (SEM) of two or three biological replicates. For statistical analysis, *p*-values were calculated using an unpaired *t*-test due to each lysin or peptide dose being compared individually to the untreated group. Additionally, to ensure clarity in presenting statistical significance, we used three types of statistical markers to highlight differences: ns, not significant, * *p* < 0.05 and a <3-log CFU reduction (with respect to the untreated group), ** *p* < 0.05 and a ≥3-log CFU reduction (with respect to the untreated group).

### 4.5. Time–Kill Assay

Using an EchoTherm chilling/heating plate, separate aliquots of both *C. acnes* strain ATCC 6919 at 10^6^ CFU/mL and P156 at 50 μg/mL in 20 mM Tris, pH 7.2, were equilibrated to 37 °C for 10 min in 1.7 mL microcentrifuge tubes. The bacteria and peptide were then combined 1:1 in a microcentrifuge tube to obtain final concentrations of 5 × 10^5^ CFU/mL and 25 μg/mL, respectively. An untreated control (bacteria without peptide) was also included. The samples were incubated for a total of 30 min at 37 °C. At 1, 5, 10, 15, 20, 25 and 30 min, an aliquot was removed. Both 100 μL directly from the sample, as well as 5 μL of each dilution from a 10-fold serial dilution, were plated on rTSB agar supplemented with 2% (*v*/*v*) glycerol in order to assess bacterial viability. The LOD was 10 CFU/mL. Error bars correspond to ±SEM of three biological replicates. For statistical analysis, *p*-values were calculated using an unpaired *t*-test between the untreated group and the peptide-treated group at each time point. ** *p* < 0.05 and a ≥3-log CFU reduction (with respect to the untreated group).

### 4.6. Cytotoxicity Assays

Healthy donor blood was collected in an EDTA-containing conical tube at The Rockefeller University Hospital. This study was approved by our Institutional Review Board, and all adult subjects provided written informed consent. hRBCs were harvested by low-speed centrifugation, washed three times, and resuspended in PBS, pH 7.4, to a 10% (*v*/*v*) concentration. Using a 96-well untreated microtiter plate, the hRBC solution was diluted 1:1 with the peptides at final concentrations from 0.5-256 μg/mL. hRBCs incubated in PBS with or without 0.1% (*v*/*v*) Triton X-100 represented positive and negative controls for hemolysis, respectively. Following a 4 h incubation at 37 °C with 5% CO_2_, intact hRBCs were pelleted at low speed, and the resulting supernatant was transferred to a new microtiter plate. Using a SpectraMax M5 microplate reader (Molecular Devices, San Jose, CA, USA), the OD_405nm_ of each supernatant was measured to quantify the relative concentration of hemoglobin released.

## 5. Conclusions

In summary, a C-terminal cationic peptide derived from PlyPi01, which is a *P. intermedia* lysin with a low antibacterial activity toward *C. acnes*, was isolated and engineered with several cationic amino acid modifications. The resulting panel of modified peptides had varying antibacterial efficacy against *C. acnes*. One peptide, P156, uniquely demonstrated a strong and rapid bactericidal activity against all tested *C. acnes* and *S. aureus* strains. Importantly, P156 remained effective under conditions relevant to acne-affected skin, including variations in temperature, salt, pH, and in combination with retinoids. These properties support P156 as a potential drug for clinical use against *C. acnes* in the context of acne vulgaris.

## Figures and Tables

**Table 1:** Engineered peptide P156 from *P. intermedia* lysin PlyPi01 with the best killing activity.

Peptide	Amino Acid Sequence	pI	MW (kDa)	Net Charge	GRAVY
P156	RRKAKAPRAEIYAQFNKWVYAGGKKLSGLVKRRRR	11.90	4.15	+11.1	−1.149

Amino acid sequence, native lysin-derived peptide (PiP01) sequence (no underline) and modified amino acids (underlined); pI, theoretical isoelectric point; MW, molecular weight; Net Charge, at pH 7.4; GRAVY, Grand Average of Hydropathicity.

**Table 2:** Bacterial strains used in this study.

Species	Strain	Source	Notes	References
*C. acnes*	ATCC 6919	ATCC	Type IA; source: facial acne	[23]
*C. acnes*	HSS B	HSS	Source: 2015 clinical isolate	This study
*C. acnes*	HSS C	HSS	Source: 2015 clinical isolate	This study
*C. acnes*	HSS D	HSS	Source: 2015 clinical isolate	This study
*C. acnes*	HSS E	HSS	Source: 2015 clinical isolate	This study
*C. acnes*	HSS F	HSS	Source: 2015 clinical isolate	This study
*C. acnes*	KPA171202	RUBC	Type IB; source: skin	[24]
*S. aureus*	8325	RUBC	MSSA; source: blood	[25]
*S. aureus*	NRS384	NARSA	USA300 pulsotype, MRSA; source: wound	[26]
*S. aureus*	MW2 BAA-1707	ATCC	USA400 pulsotype, MRSA; source: blood	[27]

ATCC, American Type Culture Collection, Manassas, VA, USA; HSS, Hospital for Special Surgery, New York, NY, USA; MRSA, methicillin-resistant *S. aureus*; MSSA, methicillin-sensitive *S. aureus*; NARSA, Network on Antimicrobial Resistance in *S. aureus*; RUBC, The Rockefeller University Bacterial Collection, New York, NY, USA.

## Data Availability

The original contributions presented in this study are included in the article. Further inquiries can be directed to the corresponding author.
